# A Ten-Day Grape Seed Procyanidin Treatment Prevents Certain Ageing Processes in Female Rats over the Long Term

**DOI:** 10.3390/nu12123647

**Published:** 2020-11-27

**Authors:** Carme Grau-Bové, Marta Sierra-Cruz, Alba Miguéns-Gómez, Esther Rodríguez-Gallego, Raúl Beltrán-Debón, Mayte Blay, Ximena Terra, Montserrat Pinent, Anna Ardévol

**Affiliations:** MoBioFood Research Group, Department of Biochemistry and Biotechnology, Universitat Rovira i Virgili, 43007 Tarragona, Spain; carme.grau@urv.cat (C.G.-B.); marta.sierra@urv.cat (M.S.-C.); alba.miguens@urv.cat (A.M.-G.); esther.rodriguez@urv.cat (E.R.-G.); raul.beltran@urv.cat (R.B.-D.); mteresa.blay@urv.cat (M.B.); ximena.terra@urv.cat (X.T.); anna.ardevol@urv.cat (A.A.)

**Keywords:** ageing, procyanidins, food intake, adiposity, glucagon/insulin, tumor

## Abstract

Adaptive homeostasis declines with age and this leads to, among other things, the appearance of chronic age-related pathologies such as cancer, neurodegeneration, osteoporosis, sarcopenia, cardiovascular disease and diabetes. Grape seed-derived procyanidins (GSPE) have been shown to be effective against several of these pathologies, mainly in young animal models. Here we test their effectiveness in aged animals: 21-month-old female rats were treated with 500 mg GSPE/kg of body weight for ten days. Afterwards they were kept on a chow diet for eleven weeks. Food intake, body weight, metabolic plasma parameters and tumor incidence were measured. The GSPE administered to aged rats had an effect on food intake during the treatment and after eleven weeks continued to have an effect on visceral adiposity. It prevented pancreas dysfunction induced by ageing and maintained a higher glucagon/insulin ratio together with a lower decrease in ketonemia. It was very effective in preventing age-related tumor development. All in all, this study supports the positive effect of GSPE on preventing some age-related pathologies.

## 1. Introduction

Adaptive homeostasis is a highly conserved process whereby cells, tissues and whole organisms transiently activate various signaling pathways in response to short-term mild internal or external perturbations, thereby resulting in transient changes in gene expression and stress resistance. There is a great deal of evidence that suggests that adaptative homeostasis declines with age. In fact, ageing is associated with a twofold detrimental impact on adaptive homeostasis [1]. Firstly, aged organisms lose their ability to rapidly modulate the adaptive homeostatic response and secondly, the compensatory basal increase in stress-responsive enzymes further compresses the maximal range of responses thus diminishing cellular ability to efficiently mitigate damage. All of this loss of adaptation leads to, among other things, chronic age-related pathologies such as cancer, neurodegeneration, osteoporosis, sarcopenia, cardiovascular disease and diabetes [2].

Metabolic derangement is one of the “seven pillars” considered among the basic mechanisms associated with age-related pathologies [3]. As glucose metabolism plays a key role in the energy management of the whole organism, its dysregulation with ageing affects several metabolic aspects [4]. Age changes in hepatic glucose output and peripheral insulin sensitivity seems to be more closely related to changes in body composition than to the ageing process itself [5]. Indeed, the intra-abdominal or visceral fat pad shows the highest association risk for diabetes mellitus, hypertension, atherosclerosis, dyslipidemia, cancers and mortality compared with peripheral obesity [6]. There is also controversy regarding the role of age in the ability of β-cells to function [5] and also its effect on β-cells although a few authors point to a higher hepatic sensitivity to plasma glucagon in older subjects [7].

Polyphenols are a broad spectrum of structures widely found in fruits and vegetables and derived products and also in beverages such as chocolate, green tea, coffee and wine [8]. They have been shown to protect against most age-related pathologies (cancer [9]; hypertension [10,11,12]; sarcopenia [13,14], neurodegeneration [15,16,17,18,19,20], osteoporosis [21,22] and cataract formation [23]). One group of these polyphenols is grape seed-derived procyanidins (GSPE), which include several flavanols, procyanidins and some phenolic acids [24]. They have been widely studied as antiobesogenic and antidiabetic agents [8,25,26], as being protective against atherogenic indexes [27] and renal failure [28] and as anti-cancer agents [29]. However, their effectiveness on ageing processes in the metabolism has not been proved. 

We have previously shown that some GSPE doses act as satiating agents in young healthy rats [30], limiting their body weight increase and adiposity [25] among other properties effective against metabolic syndrome [31,32]. Indeed, we have shown that their effects on body weight and adiposity [33] continue over the long term once the GSPE administration has finished [34]. Considering these beneficial effects of GSPE and the evidence that dietary restriction has been proven to extend lifespan [35], we hypothesize that the doses of GSPE with satiating properties may be beneficial in counteracting the ageing-induced loss of buffering in the body. We therefore aim to demonstrate this hypothesis after a 10-day GSPE oral treatment in aged rats and to observe its long term anti-ageing outcome, focusing our study on body weight and metabolism. 

## 2. Materials and Methods 

### 2.1. Proanthocyanidin Extract

The grape seed extract rich in proanthocyanidins (GSPE) came from Les Dérivés Résiniques et Terpéniques (Dax, France). According to the manufacturer, the GSPE used in this study (lot 207100) had a total proanthocyanidin content of 76.9% consisting of a mixture of monomers of flavan-3-ols (23.1%), dimers (21.7%), trimers (21.6%), tetramers (22.2%) and pentamers (11.4%).

### 2.2. Animal Model

In this study, 34 female Wistar rats were used, 10 of which were two months old (weighing 210–220 g) and 24 of which were 21 months old (weighing 300–350 g). The rats were obtained from Envigo (Barcelona, Spain). They were housed individually at a room temperature of 23 °C with a standard 12 h light-dark cycle, ventilation and ad libitum access to a standard chow diet (2014 Teklad Global 14% protein rodent maintenance diet, Envigo, Barcelona, Spain) and tap water.

### 2.3. Experimental Design

**Figure 1:**
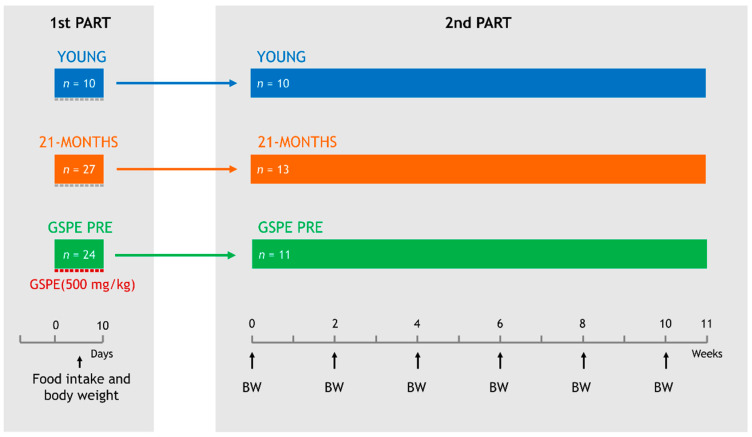
Schematic diagram of the experimental design. The experiment was divided into two parts. In the first part, the group named GSPE PRE animals were gavaged daily with a dose of 500 mg/kg of grape seed-derived procyanidins (GSPE) for 10 days while YOUNG and 21-MONTHS animals were gavaged with a vehicle. Food intake was recorded daily and body weight (BW) was measured after the 10 days of treatment. In the second part, all rats were maintained equally for 75 days and body weight was recorded every two weeks.

The experiment was divided into two parts (Figure 1). The first consisted of 10 days of treatment with GPSE by oral gavage and an evaluation of its immediate effects on food intake and body weight. The second consisted of an assessment of the long term effects of this 10-day GSPE treatment on the metabolism. All procedures were approved by the Experimental Animal Ethics Committee of the Generalitat de Catalunya, Spain (Department of Territory and Sustainability, General Directorate for Environmental and Natural Policy, project authorization code: 10183).

For the first part of the study, after a week of adaptation to the environment and another week of adaptation to oral gavage, the rats were weighed and divided into three experimental groups as follows:

(1) YOUNG, which consisted of 10 two-month-old rats.
(2) 21-MONTHS, which consisted of 27 twenty-one-month-old rats.
(3) GSPE PRE, which consisted of 24 twenty-one-month-old rats.

For 10 days, all of the animals were fasted from 15:00 h. The GSPE was dissolved in tap water and orally gavaged to the GSPE PRE animals at a dose of 500 mg GSPE/kg of body weight at 18:00 h, one hour before the dark onset. Animals in the YOUNG and 21-MONTHS groups received an equivalent volume of tap water at the same time points. The chow diet was administered at the dark onset (19:00 h). The chow intake was measured after 20 h, the next day at 15:00 h, when the animals were fasted again. At the beginning and end of the 10-day treatment, the rats were weighed. 

After the treatment, 13 animals from the 21-MONTHS group and 11 animals from the GSPE PRE group together with all of the YOUNG animals (*n* = 10) entered the second part of the study, which consisted of maintaining them for 75 more days with a chow diet and body weight records every two weeks.

### 2.4. Blood and Tissue Collection

At the end of the study, the animals were fasted for 12 h and euthanized by decapitation. The blood was collected using heparin (Deltalab, Barcelona, Spain) as anticoagulant. Plasma was obtained by centrifugation (1500 g, 15 min, 4 °C) and stored at −80 °C until analysis. The different white adipose tissue depots (retroperitoneal (rWAT), mesenteric (mWAT) and periovaric (oWAT)) and the brown adipose tissue (BAT), liver, kidneys, spleen, stomach, caecum and femur were rapidly removed, weighed, snap-frozen in liquid nitrogen and stored at −80 °C. When identified, tumorous tissues were excised and weighed. 

### 2.5. Biochemical Variables 

Commercial colorimetric enzymatic kits were used to measure levels of glucose, triacylglycerol, cholesterol, urea, creatinine (QCA, Tarragona, Spain), non-esterified fatty acids (NEFAs) (Wako, Neuss, Germany) and β-hydroxybutyrate (Ben Biochemical Enterprise, Milano, Italy) in the plasma samples in accordance with the manufacturers’ instructions. Commercial ELISA kits were used to quantify plasma levels of insulin (Millipore, Madrid, Spain) and glucagon (Mercodia, Uppsala, Sweden). 

### 2.6. Statistical Analysis 

At the end of the study, statistical analysis was performed with all collected data using one-way ANOVA with Dunnet’s post-hoc test taking the 21-MONTHS group as control. A chi-squared test was used to assess the association between tumor incidence and the variables of interest (age, treatment) in XLSTAT 2020.1 (Addinsoft, Spain) statistical software. The statistical significance for both tests was set at *p* < 0.05. 

## 3. Results

### 3.1. GSPE Reduces Food Intake and Body Weight in the Short-Term in Aged Rats 

**Figure 2:**
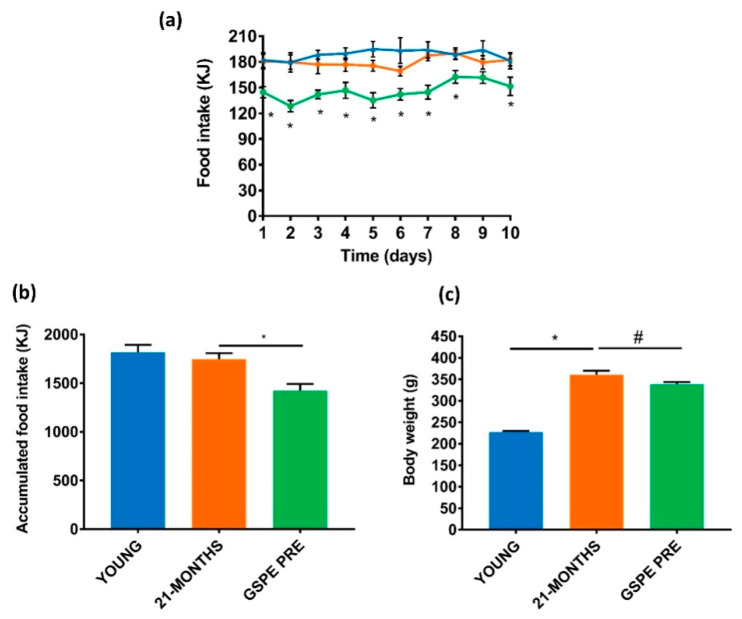
Effect of the 10-day treatment on food intake and body weight. (**a**) Daily food intake, where blue, orange and green represent YOUNG, 21-MONTHS and GSPE PRE groups, respectively. (**b**) Accumulated food intake over the 10 days of treatment. (**c**) Body weight at day 10. Values are means ± SEM. * *p*-value < 0.05, ^#^ *p*-value < 0.1 compared with 21-MONTHS rats.

Our first goal was to find out whether a dose of GSPE with satiating properties in young animals [25] was also effective in aged rats. The daily food intake was equivalent between the YOUNG and 21-MONTHS groups (Figure 2a). In agreement, the accumulated food intake for the 10 days was not different between the two groups (Figure 2b). The GSPE treatment over 10 days reduced the 20 h food intake almost daily in comparison with the 21-MONTHS rats (Figure 2a). In this case, there was a statistically significant effect on the accumulated food intake for the 10-day GSPE treatment group versus the 21-MONTHS group, as shown in Figure 2b. This reduction in the energy entering the organism over these days brought about a slightly lower body weight after the end of the treatment in the GSPE PRE rats compared with the 21-MONTHS rats (Figure 2c). As expected, the young rats showed a lower body weight than the 21-MONTHS rats (Figure 2c). 

### 3.2. The GSPE Effect on Body Weight and Adiposity Continued for Several Weeks after Administration 

**Figure 3:**
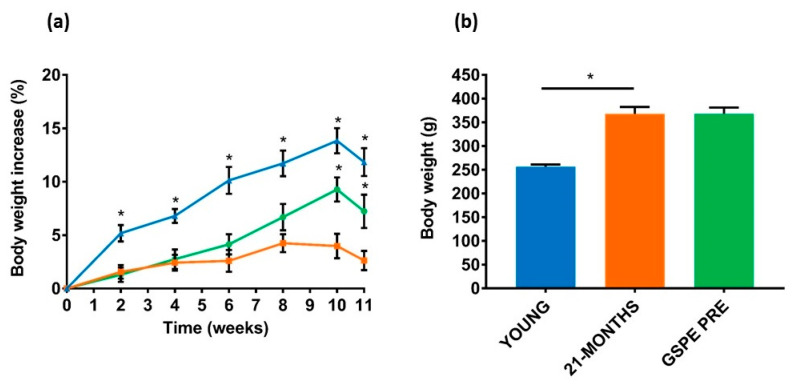
Body weight changes after GSPE pre-treatment. (**a**) Percentage of body weight increase from day 10 of the experiment. Body weight was measured once every two weeks throughout the whole experiment. Blue, orange and green represent YOUNG, 21-MONTHS and GSPE PRE groups, respectively. (**b**) Body weight at the end of the experiment. Values are means ± SEM. * *p* < 0.05 compared with 21-MONTHS rats.

Once the GSPE treatment was finished, the rats were kept for eleven weeks to evaluate the long term effects of GSPE on the 21-MONTHS rats [33]. Figure 3a shows the percentage of body weight increase during this period. The highest percentage increase was found in the young rats because they were undergoing a growth period in their lives. The 21-MONTHS rats showed a significantly lower weight increase. Initially the GSPE-treated rats showed a body weight increase similar to that of the 21-MONTHS rats (Figure 3a). However, in the tenth and eleventh weeks they significantly increased their weight to reach a final body weight close to the 21-MONTHS group (Figure 3b). 

Table 1 shows the weight of the tissues at the end of the experiment. As expected, due to their relative difference in size, all of the 21-MONTHS animals presented bigger organs than the young animals. When we compared the GSPE-treated rats with the untreated 21-MONTHS group, despite the similar body weight, the percentage of visceral adiposity was significantly lower in the GSPE PRE rats (Table 1). There were no differences in most of the other tissues except for the liver, which was also smaller in the GSPE-treated rats. The kidney showed a trend towards a lower size too. 

### 3.3. Aged GSPE Pre-Treated Rats Showed a Higher Fasting Glucagon/Insulin Ratio Eleven Weeks after the Treatment 

**Figure 4:**
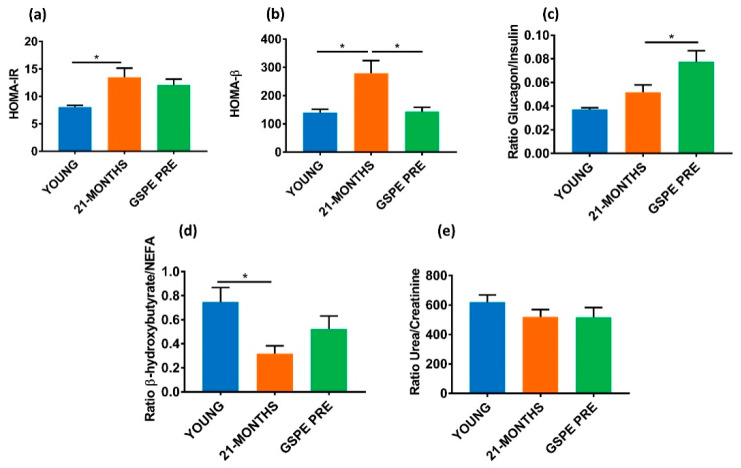
Biochemical characteristics of the groups analyzed at 13 weeks. (**a**) Insulin resistance HOMA-IR index. (**b**) Insulin resistance HOMA-β index. (**c**) Glucagon/insulin ratio. (**d**) β-hydroxybutyrate/non-esterified fatty acids (NEFA) ratio. (**e**) Urea/creatinine ratio. Values are means ± SEM. * *p* < 0.05 compared with 21-MONTHS rats.

Looking at the biochemical parameters in the plasma of fasted YOUNG and 21-MONTHS rats, glucose, non-esterified fatty acids (NEFA), urea and creatinine levels were unaffected by ageing (Table 2). A GSPE pre-treatment (GSPE PRE) showed a trend towards increasing urea. Regarding endocrine pancreas hormones, plasma insulin and glucagon were greatly increased by the ageing process (Table 2) and the GSPE pre-treatment limited the increase in the insulinemia. To gain a better picture of the metabolic status of these animals, we worked on some ratios that provided us with more information. Figure 4a shows that the GSPE pre-treatment did not avoid the increase in insulin resistance brought about by the ageing processes as indicated by the index of insulin resistance HOMA-IR. Conversely, the GSPE pre-treated group (GSPE PRE) showed a normalized pancreatic response as indicated by the lower HOMA-β of this group versus the 21-MONTHS group (Figure 4b). When we compared the glucagon/insulin ratio in the plasma of fasted animals, we found no statistically different results due to the ageing process but we did find that the GSPE pre-treated group clearly showed a higher ratio (Figure 4c). To complete the picture, Figure 4d shows that the 21-MONTHS animals produced a limited amount of β-hydroxybutyrate derived from NEFA. Finally, there were no changes in renal functionality due to ageing or GSPE pre-treatment (GSPE PRE) as defined by the urea/creatinine ratio (Figure 4e). 

### 3.4. GSPE Limits the Development of Tumors in 21-MONTHS Rats 

**Figure 5:**
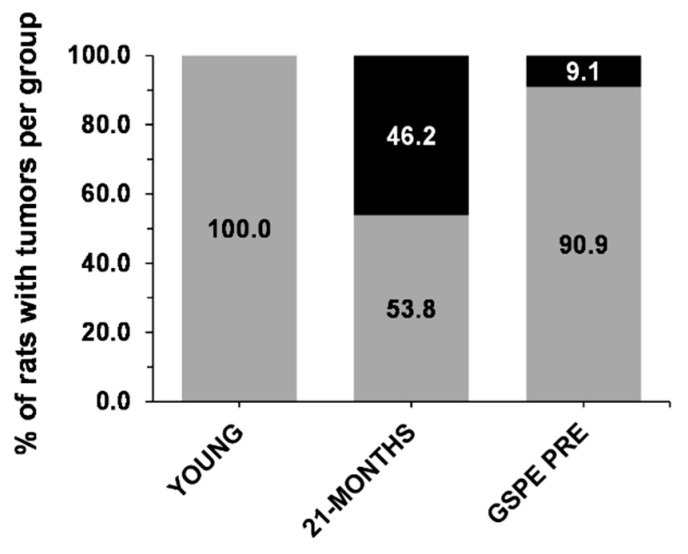
Percentage of tumors per group. Values are the percentage of rats without tumors (grey) and with tumors (black) per group studied.

One of the characteristics of ageing is an increase in the presence of tumors [36]. When the rats were dissected, all of the tumors found were counted, weighed and classified (Supplementary Table S1). Figure 5 shows that we found no tumors in the YOUNG rats but the incidence of spontaneous tumors in the 21-MONTHS rats was 46.2%. The GSPE pre-treatment limited their presence. The chi-squared test, comparing both aged groups, showed a significant reduction of the present of tumors with the GSPE pre-treatment of 9.1% (Fisher’s exact test, *p* < 0.078). 

## 4. Discussion

Ageing is a physiological process characterized by metabolic changes that lead to obesity, insulin resistance and dyslipidemia, which are risk factors for ageing-associated pathologies such as diabetes and cardiovascular disease [5,36]. GSPE has the ability to prevent several of these metabolic disruptions in young rats [33,34,37]. Here we have shown that GSPE maintains its ability in the 21-MONTHS animals. In addition, this GSPE treatment showed a trend to prevent the development of tumorigenic tissue growths, also closely associated with the ageing processes. 

In previous studies, we defined a dose of 500 mg/kg GSPE as responsible for reducing food intake and body weight in young rats [25]. Here we have found a similar effect on food intake in aged animals over the 10 days that the treatment lasted. In this case, the food intake reduction of around 20% had only a slight effect on body weight immediately after the treatment because these animals had already reached adulthood and their body weight remained constant. In young rats, studies on a caloric restriction of 20% have shown reductions in body weight increase of around 40% in fifteen days [25,38]. In 21-month-old animals, the same caloric restriction takes several weeks to obtain a 30% decrease in body weight [35]. We found that in young rats the effects of GSPE were maintained for several weeks after the treatment had finished [33]. We have now shown that in aged rats, the GSPE effect limiting body weight increase was also maintained for eight more weeks after the end of the treatment. This was probably due to the ability of proanthocyanidins to limit adipose accrual several weeks after the end of the treatment [34]. Indeed, we also found that GSPE was able to maintain a lower percentage of visceral adiposity 11 weeks after the treatment. The effects of this GSPE dose as a preventive works in a similar way to caloric restriction interventions that reverse ageing-associated visceral fat increase and have an important impact on decreasing insulin resistance [39]. Our results eleven weeks after the end of the treatment with GSPE showed a preventive effect on the increase in the HOMA-β index as also found in some other models of caloric restriction [40]. However, it did not show any clear protective effect on the insulin resistance index (HOMA-IR). This lack of effect of GSPE on insulin resistance could be the explanation for the dyslipidemic profile found in the GSPE pre-treated group at the end of the study. There is a definite cause/effect associated with insulin resistance on muscle and adipose tissue [41] and dyslipidemia [42]. Dyslipidemia, together with a decreased ketosis, has also been observed in recent work with 24-month-old male Wistar rats [43,44] and is in line with what we saw in our 21-month-old female rats. There have been some controversial results regarding caloric restriction effects on ketone bodies. A lower ability with ageing to synthetize ketone bodies in the intestine has been reported [45] as has a lower consumption of ketone bodies in the kidney [46]. In both examples, these reductions were reverted by caloric intake restriction. We previously found a higher expression of HMGCS2 in the liver of young rats five weeks after the last GSPE dose [34] suggesting a greater ability in the GSPE-treated rats to produce ketone bodies. In the present study, we did not have a statistically different effect on plasma β-hydroxybutyrate but this could be due to the length of the study.

In a fasting situation when blood insulin is low, glucagon levels are high and therefore fat oxidation and ketosis are increased and hepatic lipogenesis is activated [47]. Glucagonemia was also increased in our 21-MONTHS rats as found by Fernández et al. [44], although there is no wide consensus on glucagonemia and ageing [7]. Here, GSPE brought about a situation different than the 20% caloric restriction of Fernández et al. [44]. This higher glucagonemia in GSPE pre-treated rats clearly produced a higher glucagon/insulin ratio despite the weeks without treatment. This could explain the trend found in the limited increase in visceral adiposity in these animals. A higher presence of glucagon versus insulin during a fasting situation favors higher fat oxidation [48], which together with a higher liver sensitivity to glucagon on ageing [7] would produce a higher hepatic gluconeogenesis. We did not find a statistical change on glycemia in these GSPE pre-treated rats sacrificed in a fasting situation, but we cannot discard that it happened at some point earlier than the several weeks post-treatment when we were measuring it. 

One of the significant organ systems that declines with ageing is the kidney. Changes in renal structure (reduction in mass) and function (glomerular filtration rate (GFR)) accompany advancing age [49]. Here, we found no effect of age on kidney size nor did we find any on urea, creatinine or their ratio. However, we did observe a GSPE trend after several weeks towards a smaller kidney size and an increase in urea versus the 21-MONTHS group but without any significant change in the urea/creatinine ratio, suggesting the maintenance of the glomerular filtration rate. A similar dose of 500 mg/kg GSPE had beneficial effects on reducing induced acute renal injury and chronic kidney fibrosis in young mice [50] and diabetic-associated renal injury in young rats [28]. Our results therefore suggest no clear preventive effects on kidney functionality. 

All in all, this GSPE pre-treatment produced a long term effect close to a caloric restriction state in the rats. Its preventive effect against tumorigenesis was also observed. Caloric restriction prevents tumorigenesis by decreasing the metabolic rate and oxidative damage [51]. Here, we found spontaneous tumors on 46.2% of the 25-month-old rats at the end of the study, a value that was lower than the reported number of age-associated tumors on female Wistar rats [52]. With the sample size used in this study, the GSPE treatment showed a trend towards a lowered incidence of tumors on GSPE PRE rats to 9.1%. A bigger sample size would be necessary to fully demonstrate this effect. Related to the possible reasons involving caloric restriction, the GSPE tumor suppressing effect may be explained by its modulation of antiproliferative and proapoptotic genes [53] such as the tumor suppressing factor p53 [54] and NF-κβ [55] observed in different cancerous cell lines, their anti-inflammatory properties [56] and antioxidant properties [57]. 

## 5. Conclusions

We can conclude that GSPE showed interesting properties on 21-MONTHS rats. It acted to limit food intake resulting in a decreased body weight after treatment. Eleven weeks after the treatment, GSPE maintained its effects on limiting visceral adipose tissue growth, prevented the increase in the HOMA-β index and maintained a higher glucagon/insulin ratio together with a reduced incidence of age-associated spontaneous tumors. A few of these effects might be related to their caloric restriction mimetic effect.

## Figures and Tables

**Table 1:** Morphometric characteristics at the end of the experiment (week 11).

Variable	YOUNG	21-MONTHS	GSPE PRE
*n*	10	13	11
Body weight (g)	256.6 ± 4.3 *	367.4 ± 15.0	366.8 ± 14.2
mWAT (g)	3.7 ± 0.2 *	13.1 ± 1.2	10.5 ± 1.3
oWAT (g)	6.8 ± 0.2 *	16.6 ± 1.5	15.5 ± 1.5
rWAT (g)	4.0 ± 0.3 *	11.1 ± 1.1	10.6 ± 1.1
Total visceral WAT (g)	14.6 ± 0.2 *	39.5 ± 3.4	34.8 ± 3.3
BAT (g)	0.4 ± 0.0 *	0.7 ± 0.1	0.7 ± 0.1
% visceral adiposity	5.4 ± 0.2 *	11.3 ± 0.6	9.5 ± 0.7 *
Liver (g)	6.2 ± 0.2 *	8.7 ± 0.4	7.7 ± 0.2 *
Spleen (g)	0.5 ± 0.0 *	0.8 ± 0.0	0.8 ± 0.0
Kidney (g)	0.8 ± 0.0 *	1.0 ± 0.0	0.9 ± 0.0 ^#^

mWAT: mesenteric white adipose tissue; oWAT: periovaric white adipose tissue; rWAT: retroperitoneal white adipose tissue; BAT: brown adipose tissue; total visceral WAT: sum of all white adipose tissues. Values are means ± SEM. * *p* < 0.05 compared with 21-MONTHS rats. Trends: ^#^ *p* < 0.1 compared with 21-MONTHS rats.

**Table 2:** Plasma biochemical characteristics at the end of the experiment (week 11).

Variable	YOUNG	21-MONTHS	GSPE PRE
Plasma			
Glucose (mM)	7.3 ± 0.3	7.0 ± 0.3	8.1 ± 0.6
TAG (mM)	0.4 ± 0.1 ^#^	0.6 ± 0.1	0.5 ± 0.1
NEFA (mM)	1.0 ± 0.1	1.0 ± 0.1	0.9 ± 0.1
Cholesterol (mM)	2.6 ± 0.1 *	4.5 ± 0.4	4.2 ± 0.4
β-Hydroxybutyrate (mM)	0.7 ± 0.1 *	0.3 ± 0.1	0.5 ± 0.1
Urea (mM)	4.2 ± 0.2	3.8 ± 0.2	4.3 ± 0.1 ^#^
Creatinine (μM)	7.1 ± 0.3	7.7 ± 0.6	7.3 ± 0.7
Insulin (pM)	182.7 ± 1.0 *	322.2 ± 36.4	233.7 ± 13.4 *
Glucagon (pM)	7.2 ± 1.3 *	18.2 ± 2.5	18.8 ± 1.9

Values are means ± SEM. * *p* < 0.05 compared with 21-MONTHS rats. Trends: ^#^ *p* < 0.1 compared with 21-MONTHS rats. TAG: triglycerides; NEFA: non-esterified fatty acids.

## Supplementary Materials

The following are available online at https://www.mdpi.com/2072-6643/12/12/3647/s1, Table S1: Number, weight and classification of tumor per group.
