# Supplementary material for: A Ten-Day Grape Seed Procyanidin Treatment Prevents Certain Ageing Processes in Female Rats over the Long Term
Source: Nutrients. 2020 Nov 27;12(12):3647. doi: 10.3390/nu12123647 (PMC7759988; doi:10.3390/nu12123647)
Supplement: Supplementary file 1 [file nutrients-12-03647-s001.pdf]

A ten-day grape seed procyanidin treatment prevents certain ageing processes in female rat over the long term

Carme Grau-Bové *et al.*

Supplementary material

Supplementary table S1. Number, weight and classification of tumour per group. Number of animals with tumours are shown in total units and percentage in parenthesis. Weight of tumours per group is shown as mean ± SEM. The tumours found in these rats were located in ovaries or fallopian tubes, subcutaneous, hypophysis and pancreas. The number of animals with each of these types of tumour are shown in total units and percentage in parenthesis.

Group	Total animals (n)	Animals with tumour (n and %)	Weight (g)	Type of tumor (n and %)				
				Ovaries and Fallopian tubes	Intestine	Subcutaneous	Hypophysis	Pancreas
Young	10	0 (0.0%)	0.0 ± 0.0	0 (0.0%)	0 (0.0%)	0 (0.0%)	0 (0.0%)	0 (0.0%)
21-MONTHS	13	6 (46.2%)	2.1 ± 1.4	2 (15.4%)	1 (7.7%)	2 (15.4%)	0 (0.0%)	1 (7.7%)
GSPE PRE	11	1 (9.1%)	0.01 ± 0.001	0 (0.0%)	0 (0.0%)	0 (0.0%)	1 (9.1%)	0 (0.0%)
